# tRNA ^Arg^ binds *in vitro* TDP-43 RNA recognition motifs and ligand of Ate1 protein LIAT1

**DOI:** 10.17912/micropub.biology.001224

**Published:** 2024-07-15

**Authors:** Ljiljana Sjekloća, Emanuele Buratti

**Affiliations:** 1 Molecular Pathology, International Centre for Genetic Engineering and Biotechnology, Padriciano 99, Trieste 34149, Italy

## Abstract

Transactive response DNA-binding protein 43 (TDP-43) is important for RNA metabolism in all animals and its malfunctions are linked to neurodegenerative and myodegenerative diseases in humans. Arginyl transferase Ate1 transfers an arginyl group from arginylated tRNA ^Arg^ to proteolytic fragments of the C-terminal region of TDP-43, prompting their degradation by the ubiquitin proteasome system, thus contributing to TDP-43 proteostasis. To gain more insight into the molecular basis of TDP-43 arginylation, we tested if tRNA ^Arg^ could bind *in vitro* to a panel of recombinant multidomain constructs of human TDP-43 or to the arginylation cofactor protein LIAT1. We observed that *in vitro-* transcribed human tRNA ^Arg^ directly interacts with the RNA recognition motifs of TDP-43 and that their binding is stabilized by dimerization, which is promoted by the amino-terminal domain and the nuclear localization signal sequence of TDP-43. Moreover, the same human TDP-43 constructs that bind tRNA ^Arg^ bind native fungal tRNA ^Phe^ , suggesting that TDP-43 can bind different populations of tRNAs. Interestingly, human tRNA ^Arg^ is also able to bind recombinant mouse LIAT1 suggesting, for the first time, that LIAT1 is an RNA-binding protein. Our findings open a new perspective on the intricate crosstalk between protein and tRNA metabolism, which may eventually contribute to the understanding of the role of TDP-43 proteostasis in health and disease.

**Figure 1. In vitro -transcribed human tRNA Arg binds human TDP-43 RNA recognition motifs and full-length mouse LIAT1:**
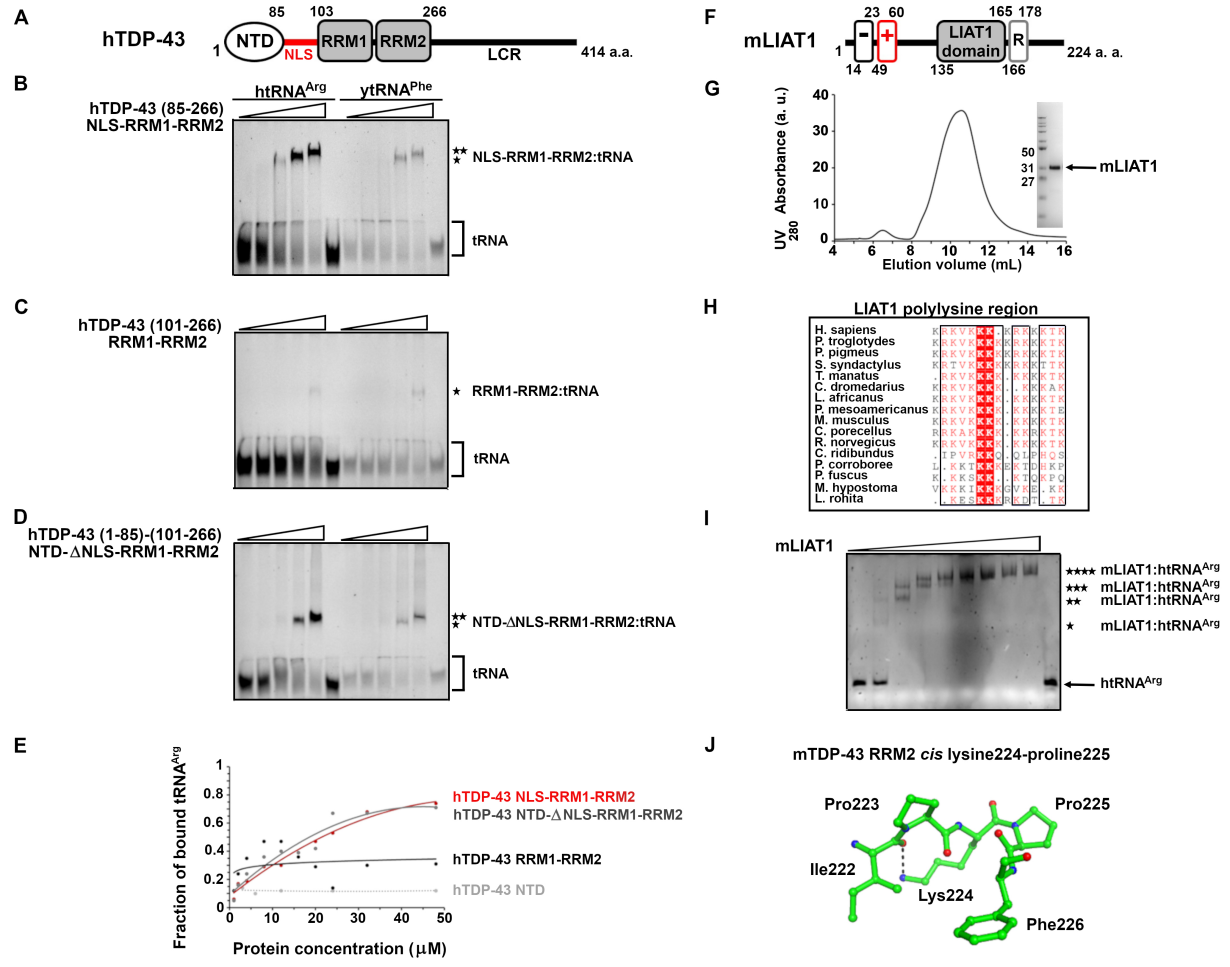
( **A** ) Schematic representation of human TDP-43: NTD- amino terminal domain, NLS- nuclear localization sequence, RRM – RNA recognition motif, LCR- low complexity region. ( **B** , **C** , **D** , **E** ) EMSA assays to asses *in vitro* interaction of fixed amount of human *in vitro-transcribed* tRNA ^Arg^ (left half of PAGE) or native yeast tRNA ^Phe^ (right half of PAGE) with increasing amounts of recombinant fragments of human TDP-43: ( **B** ) hTDP-43 NLS-RRM1-RRM2, ( **C** ) hTDP-43 RRM1-RRM2, ( **D** ) hTDP-43 NTD-ΔNLS-RRM1-RRM2, ( **E** ) quantitation of htRNA ^Arg^ binding to hTDP-43 fragments performed by gel image analysis. ( **F** ) Schematic representation of mouse LIAT1: “-“ - poly glutamate region, “+” - polylysine region, LIAT1 domain, R - LIAT1 repeat. ( **G** ) Size exclusion Superdex 75 HR10/30 chromatogram of mLIAT1 and SDS-PAGE gel of eluted mLIAT1. ( **H** ) Multiple sequence alignment of full-length vertebrate LIAT1 proteins shown in correspondence of polylysine region. ( **I** ) EMSA assay to asses *in vitro* interaction of fixed amount of htRNA ^Arg^ with full length recombinant mLIAT1; asterisks denote different migration of mLIAT1:htRNA ^Arg^ complexes.( **J** ) Ball-and-stick representation of *cis* peptide bond between mTDP-43 Lys224 and Pro225 and of interaction between Ile222 and Lys224 in crystal structure of mouse TDP-43 (mTDP-43) RRM2 (PDB 3D2W).

## Description

Post-translational Nt-arginylation is the non-ribosomal transfer of an arginyl group from arginylated tRNA ^Arg^ to target proteins that bear an amino terminal aspartate, glutamate or oxidized cysteine. This modification occurs in all eukaryotes and is catalyzed by the arginyl-tRNA ^Arg^ protein transferase Ate1 (Manahan and App, 1973; Balzi et al., 1990; Hu et al., 2006) . Nt-arginylation may direct proteins to degradation by the ubiquitin proteosome system (Ciechanover and Kwon, 2015; Varshavsky, 2019) and by autophagy (Jiang et al., 2016) , or it may influence protein dimerization and intracellular localization (Sambrooks et al., 2012). Nt-Arginylation has neuroprotective and neuroregenerative effects (Bongiovanni et al., 1995; Ingoglia, 2015; Ingoglia and Jalloh, 2016) and has been shown to influence the folding and proteostasis of neuronal proteins important in human neurodegeneration such as amyloid beta, synuclein and TDP-43. In particular, TDP-43 is important for RNA splicing and regulation of translation (Freibaum et al., 2010; Buratti and Baralle, 2012; Bhardway et al., 2013) . In humans, TDP-43 is involved in fatal neurodegenerative diseases such as amyotrophic lateral sclerosis (ALS) and frontotemporal lobar degeneration FTLD (Neumann et al., 2006) , as well as in myodegenerative diseases such as sporadic inclusion bodies myositis sIBM (Cortese et al., 2014) and myoclonic epilepsy associated with ragged red fibers MERRF (Mancuso et al., 2004; Mori et al., 2019) . Upon prolonged cellular stress, TDP-43 translocates from the nucleus and becomes increasingly cytoplasmic, proteolytically fragmentated, hyperphosphorylated and polyubiquitinated, and aggregates in insoluble cytoplasmic inclusions. Progranulin mediates caspase-dependent fragmentation of TDP-43 generating a heterogenous population of C-terminal fragments (CTFs) of ~ 25 kDa and ~35 kDa that have been found in the cytoplasmic inclusions of some FTLD and ALS patients as well as in model animals (Neuman et al., 2006; Zhang et al., 2007; Igaz et al., 2008; Li et al., 2015) . A decrease in Nt-arginylation of the CTFs of human TDP-43 has been shown to increase their aggregation in the cytoplasm (Brower et al. 2013; Kasu et al. 2018) which may contribute to the imbalance of intracellular TDP-43 and related proteinopathy.

At a molecular level, TDP-43 is a dimeric protein prone to oligomerization (Mompean et al., 2017; Afroz et al., 2017; Wang et al., 2018) and it consists of a conserved region composed of an amino-terminal dimerization domain (NTD), a nuclear localization signal (NLS) and two tandem RNA recognition motifs RRM1 and RRM2 followed by a non-conserved glycine-rich carboxyl terminus of low structural complexity (Laurents et al., 2021; Capitini et al., 2021; Figure 1A ). To further the thorough understanding of the molecular basis of TDP-43 Nt-arginylation, we used an *in vitro* system to transcribe human tRNA ^Arg^ containing also a 3’ CCA trinucleotide (htRNA ^Arg^ ) and tested its interaction with a set of recombinant constructs of human TDP-43 (hTDP-43) consisting of one or more of the features in the conserved region. The constructs produced were hTDP-43 NTD (residues 1-85), NLS-RRM1-RRM2 (residues 85-266), RRM1-RRM2 (residues 101-266), and a truncated construct NTD-ΔNLS-RRM1-RRM2 (residues 1-85 and 101-266) lacking the NLS linker because it is a hotspot for proteolytic attacks by host proteases. NTD, NTD-ΔNLS-RRM1-RRM2 and NLS-RRM1-RRM2 were purified as dimeric proteins, whilst RRM1-RRM2 was monomeric. When tested *in vitro* for binding to htRNA ^Arg^ , we detected stable complex formation with NLS-RRM1-RRM2 ( Figure 1B Left ), RRM1-RRM2 ( Figure 1C Left ) and NTD-ΔNLS-RRM1-RRM2 ( Figure 1D Left ). We did not observe that hTDP-43 NTD on its own could form a stable complex with htRNA ^Arg^ . The hTDP-43 NLS-RRM1-RRM2:htRNA ^Arg^ complex was very stable, unaffected by doubling the physiological concentration of KCl or by high concentrations (up to 20%) of PEG 8000 used as a macromolecular crowding agent. The RRM1-RRM2 complex with htRNA ^Arg^ contained less htRNA ^Arg^ than the one with NLS-RRM1-RRM2 ( Figure 1E ). Besides binding to htRNA ^Arg^ we also observed successful binding of these constructs to native yeast tRNA ^Phe^ (ytRNA ^Phe^ ; Figure 1B, 1C, 1D Right), suggesting that general tRNA binding could be a property of the hTDP-43 central region. Increasing the concentration of hTDP-43 NLS-RRM1-RRM2 or NTD-ΔNLS-RRM1-RRM2 resulted in higher band shifts of both htRNA ^Arg^ and ytRNA ^Phe^ ; such migration pattern could be explained by protein or RNA concentration-dependent oligomerization and/or formation of different types of protein:tRNA complexes.

Mammalian Ate1 co-factor protein LIAT1 can enhance *in vitro* Nt-arginylation (Brower et al., 2014) . Vertebrate LIAT1 proteins consist of conserved amino terminal and central regions which contain a glutamate-rich region, a lysine-rich region, and a ~30 residue-long Ate1-binding region termed the LIAT1 domain, and a variable carboxyl-terminal region (Brower et al., 2014) . Sequence analysis suggested that both human and mouse LIAT1 are intrinsically disordered proteins (Brower et al., 2014; Arva et al., 2020) as supported by their AlphaFold models, AF-Q6ZQX7 and AF-Q810M6 (Jumper et al., 2021) . We checked if LIAT1 could bind htRNA ^Arg^ . For interaction assays, we opted to use mouse LIAT1 ( Figure 1F ) because it is the shortest mammalian LIAT1 that was shown to interact directly with Ate1 (Brower et al., 2014; Arva et al., 2021) . Recombinant mouse LIAT1 (mLIAT1) produced in *Escherichia coli* , purified in aerobic conditions as a soluble light brown protein. The construct eluted in size exclusion chromatography as a ~48 kDa protein in agreement with the theoretical molecular weight of dimeric mouse LIAT1 ( Figure 1G ). mLIAT1 migrated on SDS-PAGE gels as a ~30 kDa protein ( Figure 1G ) which is bigger than expected for a protein of ~24 kDa theoretical molecular weight. This anomalous migration might be explained by the presence of a ligand, intramolecular disulfide bridges, detergent binding or by being intrinsically disordered. Sequence analysis of mouse LIAT1 by HeMoQuest (Paul George et al., 2020) predicts the existence of a transient heme-binding site (residues Cys122 and Cys197) and the presence of disulfide bonds. The latter is supported by the presence of a CXXC motif (Cys194 and Cys197), which in some redox proteins regulates disulfide bond formation and dimerization (Chievers et al., 1996). These predictions could explain the brownish color of mLIAT1, as well as its oligomerization and anomalous migration in SDS-PAGE. We note that mouse LIAT1 also contains a putative bipartite nuclear localization signal sequence (amino acids K ^49^ RKVKKKKKKKKTKG ^63^ ) that is conserved in different vertebrate LIAT1 proteins ( Figure 1H ). The presence of this signal could explain a previous observation of mouse LIAT1 being found in both the nucleus and the cytosol (Arva et al., 2021) . We observed *in vitro* interaction between mLIAT1 and htRNA ^Arg^ resulting in four different protein concentration-dependent mLIAT1:htRNA ^Arg^ complexes ( Figure 1I ). This result suggests the coexistence of different mLIAT1 oligomers which bind separately to htRNA ^Arg^ , and this is in accordance with a previous study that reported LIAT1 self-oligomerization *in vivo* (Bower et al., 2014).

We could not analyze if the full-length human TDP-43 or its CTFs, human LIAT1 or human Ate1 interact with htRNA ^Arg^ , because of problems encountered in the recombinant production of these constructs. These difficulties may be due to unmet folding requirements. In this regard, we note that in both the three-dimensional structures of mouse TDP-43 RRM2, PDB 3D2W (Kuo et al., 2009) and of yeast ATE1, PDB 7WG4 (Kim et al., 2022) , there is a *cis* prolyl peptide bond ( Figure 1J ). In the future, it would be of interest to identify if and which *trans-cis* peptidylprolyl isomerases are required for successful human TDP-43 and Ate1 folding.

In summary, human TDP-43 multidomain fragments that include the RRMs were observed to bind efficiently to *in vitro-* transcribed human tRNA ^Arg^ . *In vivo,* TDP-43 CTFs are heterogenous in length, and often contain parts of the RRMs - e.g. TDP-43 CTF 219-414 and 247-414 (Bower et al., 2013; Li et al., 2015; Kasu et al., 2018). These RRM-containing CTFs might interact with tRNA ^Arg^ , influencing binding specificity and Nt-arginylation kinetics and contributing to the self-regulation of TDP-43 proteostasis and pathology. We also observed that the RRM-containing constructs of TDP-43 that bind tRNA ^Arg^ are also able to bind to native fungal tRNA ^Phe^ . The interaction between TDP-43 and tRNA ^Phe^ could be of physiological relevance given that a point mutation of human tRNA ^Phe^ causes MERRF myopathy characterized by cytoplasmic inclusions containing TDP-43 (Mancuso et al., 2004) and that aminoacylation of tRNA ^Phe^ is compromised in ALS patients (Malnar Črnigoj et al., 2023). Moreover, a previous report showed that *in vivo* recombinant polyhistidine-tagged human TDP-43 can also bind mitochondrial tRNA ^Asn^ , tRNA ^Gln^ and tRNA ^Pro^ (Izumikawa et al., 2017) . Taken together, this report and our data suggest that TDP-43 may be involved in the metabolism of tRNAs in general. Finally, we observed strong binding of recombinant mouse LIAT1 to htRNA ^Arg^ , a capacity that could extend to other LIAT1 sequelogues. In addition to binding to Ate1, mouse LIAT1 interacts with bifunctional arginine demethylase and lysine hydroxylase jumonji domain-containing protein 6 (JMJD6). JMJD6 is involved in RNA splicing and, together with ribosomal proteins RPS14 and RPS19, in tRNA binding and ribosome biogenesis (Bower et al., 2014; Arva et al, 2021). Noteworthy, a jumonji domain-containing hydroxylase protein TYW5 binds and modifies human tRNA ^Phe^ (Noma et al., 2010; Kato et al., 2011) . Given that the partner proteins of LIAT1 are involved in different aspects of RNA metabolism, it is foreseeable that LIAT1 capacity to bind RNA could have functional implications which may reflect also on TDP-43 proteostasis.

## Methods

Synthetic genes encoding *Homo sapiens* TDP-43 (UniprotKB entry Q13148) or *Mus musculus* LIAT1 (UniProtKB- Q810M6) were used for PCR production of DNA amplicons encoding full length multidomain constructs of the conserved region of human TDP-43 (hTDP-43) constructs and mouse LIAT1 (mLIAT1) which were then cloned in pET-LIC vectors 2B-T, 2Bc-T, 2T-T and 2TcT conferring an amino-terminal His _6_ , carboxyl-terminal His _6_ , amino terminal His _6_ -thioredoxin, or carboxyl-terminal His _6_ - thioredoxin tags (respectively), and a TEV cleavage site, transformed in *Escherichia coli* BL21 KRX cells (Promega) then grown in Terrific Broth medium supplemented with 100 μg L ^-1^ ampicillin at 37°C until OD _600_ ~ 0.6 when recombinant protein expression was induced with 1mM IPTG and 0.2 % rhamnose for 12 hr at 20°C. Cells were lysed by sonication in 1 M NaCl, 5% glycerol, 50 mM Hepes-NaOH pH 7.8, 2 mM 2-mercaptoethanol supplemented with 1 mM PMSF, 5 mM pepstatin and 5 mM leupeptin. Recombinant proteins were applied on nickel nitrilotriacetic acid affinity NiNTA resin (Qiagen), extensively washed with lysis buffer and eluted in 150 mM NaCl, 200 mM imidazole, 5% glycerol, 50 mM Hepes pH 7.7, 2 mM 2-mercaptoethanol. Eluted proteins were concentrated in centrifugal filter devices with cut-off 3 kDa or 10 kDa (PALL) and buffer exchanged on size exclusion chromatography column HiLoad pg 16/60 equilibrated in 150 mM KCl, 20 mM NaCl, 5% glycerol, 1 mM MgCl _2_ , 50 mM Hepes-KOH 7.5, 2 mM 2- mercaptoethanol. Recombinant tags were removed by digestion with TEV protease, digestion reaction was applied on NiNTA resin and the flow through containing tagless protein was concentrated to ~ 200 μM and loaded on HiLoad Superdex 75 pg 10/30 column (Cytiva) in 150 mM KCl, 20 mM NaCl, 1 mM MgCl _2_ , 5% glycerol, 50 mM Hepes-KOH pH 7.7, 2 mM 2- mercaptoethanol. For all interaction essays we used only recombinant proteins without any tag which purity and identity were checked by SDS-PAGE (NuPAGE Bis-Tris 4-12%, ThermoFisher) and by mass spectrometry.

Synthetic gene encoding full length human arginine transfer RNA bearing anticodon ACG (transfer RNA data base entry tdbD00002599, tRNA ^Arg^ ACG) and 3’ end CCA extension was placed under T7 promoter (TAATACGACTCACTATA) in pEX-A128 plasmid (Eurofins) and used as template for 1 mL PCR reaction in: 2 mM dNTP each, DNA Taq polymerase (NEB) in respective 1x PCR reaction buffer (NEB), 1 μM forward primer recognizing T7 promoter, 1 μM reverse primer recognizing 3’ end of the tRNA ^Arg^ -CCA encoding gene; resulting amplicon was used as input for 25 mL *in vitro* transcription reaction containing: 5 mM rNTP each (Jena Bioscience), 25 mM MgCl _2_ , 2 mM spermidine (Sigma-Aldrich), 30 mM TRIS pH 8.0, 20 mM dithiothreitol, 0.01 % triton X-100, 20 units of yeast inorganic pyrophosphatase (Roche), and ~ 0.1 mg mL ^-1^ recombinant polyhistidine-tagged T7 RNA polymerase. After 24 hours incubation at 37°C, transcription mixture was supplemented with 1 mM CaCl _2_ and incubated with 150 units of RNase-free DNase RQ1 (Promega) for 30 min, at 37°C. Transcription reaction was then centrifuged at 3500 rpm for 5 min and applied on two HP Q columns (Cytiva) connected in series equilibrated in solution A (50 mM Na _2_ PO _4_ pH 6.5, 150 mM NaCl, 0.2 mM EDTA); produced RNA was separated from other components of transcription mixture by an increasing gradient of NaCl obtained by FPLC mixing at room temperature (20° C) solution A and solution B (50 mM Na _2_ PO _4_ , 1M NaCl, 0.2 mM EDTA), and all eluted peak fractions were analyzed on 8M, 10% polyacrylamide, TRIS/borate/EDTA gels ran in 1x TBE (130 mM TRIS, 45 mM boric acid, 2.5 mM EDTA). Fractions containing htRNA ^Arg^ -CCA were concentrated in 3 kDa cut-off centrifugal device (Pall) and applied on HiLoad Superdex 75 16/60 column (Cytiva) equilibrated in size exclusion SEC buffer: 150 mM KCl, 20 mM NaCl, 1 mM MgCl _2_ , 5% glycerol, 50 mM HEPES pH 7.7. *In vitro* -transcribed htRNA ^Arg^ -CCA eluted in two peaks corresponding to dimer and monomer; monomeric htRNA ^Arg^ -CCA was quantitated assuming an extinction coefficient ε = 734 600 M ^-1^ cm ^-1^ at 260 nm and used without being further concentrated. In text and in figures *in vitro* -transcribed htRNA ^Arg^ -CCA is abbreviated as htRNA ^Arg^ .

Yeast phenylalanine tRNA (ytRNA ^Phe^ ) was purified from yeast (Merck) and resuspended to 1 mg mL ^-1^ in SEC buffer, renatured (treated 65°C for 90 seconds and then allowed to slowly cool down to room temperature inside lab heating block) and applied on a HiLoad Superdex 75 16/60 column in SEC buffer; eluted peak fractions were quantified assuming an extinction coefficient ε = 560 000 M ^-1^ cm ^-1^ at 260 nm.

Protein and RNA molecular size was assessed by size exclusion chromatography SEC applying 500 μL of protein sample (> 95% purity, in SEC buffer) on Superdex 75 pg 10/30 column (Cytiva) in SEC buffer and ran at 0.5 mL min ^-1^ ; the calibration curve for the used column was obtained applying separately 500 μL of: myoglobin (150 kDa), polyhistidine-tagged T7 RNA polymerase (97 kDa), bovine serum albumin (66 kDa), ovalbumin (43 kDa), chimotripsinogen (25.9 kDa), and polyhistidine-tagged thioredoxin (15 kDa).

Electromobility shift assays (EMSA) were performed on 6% polyacrylamide gels (1 mm x 100 mm x 80 mm), in 0.5 x TBE, at 80V, 20°C, for 70 min. 30 μL reactions were set mixing increasing amounts of recombinant protein (0.5 - 32 μM final concentration in reaction, assuming total protein concentration as of monomeric protein) with fixed amount of tRNA (0.5 μM final concentration in reaction) in SEC reaction buffer supplemented with 5% PEG 8000, adding tRNA as the last component and incubation time was 30 min at 20°C, after what native gel loading buffer (2.5xTBE, 50 % glycerol, 0.1 % blue bromophenol, 0.1 % xylene cyanol) was added and 15 μL of sample were loaded. Gels were stained for 15 min with nucleic acids binding chelating agent SYBR safe (EuroClone) diluted in 0.5xTBE, washed in 0.5xTBE and visualized on UV _260_ transilluminator. ImageJ (NIH) was used to quantify unbound fraction of loaded tRNA and plotted using Excel; the fraction of bound tRNA was obtained by dividing difference of total loaded tRNA and unbound tRNA with total tRNA.

PyMol (DeLano, 2020) was used for 3D models visualization and analysis and for generating structural figure; Clustal Ω (Sievers et al., 2012) was used for multiple sequence alignment; Motif Scan (Gasteiger et al., 2003) was used for protein sequence analysis.
