# Corrigendum: tRNA ^Arg^ binds *in vitro* TDP-43 RNA recognition motifs and ligand of Ate1 protein LIAT1

**DOI:** 10.17912/micropub.biology.001527

**Published:** 2025-01-31

## Description

The authors, Sjekloća, Lj.; and Buratti, E., submit the following correction of the figure.

In the version of this article originally published, in Figure 1F mLIAT1 carboxyl terminal residue number 228 was mislabeled as 224.

In Figure 1F, mLIAT1 carboxyl terminal residue number 224 should be corrected to 228.

This error has been corrected in the HTML and PDF version of the article.

